# Evaluation of two-point Dixon water-fat separation for liver specific contrast-enhanced assessment of liver maximum capacity

**DOI:** 10.1038/s41598-018-32207-6

**Published:** 2018-09-14

**Authors:** Michael Haimerl, Ute Probst, Stefanie Poelsterl, Claudia Fellner, Dominik Nickel, Kilian Weigand, Stefan M. Brunner, Florian Zeman, Christian Stroszczynski, Philipp Wiggermann

**Affiliations:** 1 Department of Radiology, University Hospital Regensburg, Regensburg, Germany; 2 MR Applications Predevelopment, Siemens Healthcare GmbH, Erlangen, Germany; 3 Department of Internal Medicine I, University Hospital Regensburg, Regensburg, Germany; 4 Department of Surgery, University Hospital Regensburg, Regensburg, Germany; 5 Center for Clinical Trials, University Hospital Regensburg, Regensburg, Germany

## Abstract

Gadoxetic acid-enhanced magnetic resonance imaging has become a useful tool for quantitative evaluation of liver capacity. We report on the importance of intrahepatic fat on gadoxetic acid-supported T1 mapping for estimation of liver maximum capacity, assessed by the realtime ^13^C-methacetin breathing test (^13^C-MBT). For T1 relaxometry, we used a respective T1-weighted sequence with two-point Dixon water-fat separation and various flip angles. Both T1 maps of the in-phase component without fat separation (T1_in) and T1 maps merely based on the water component (T1_W) were generated, and respective reduction rates of the T1 relaxation time (rrT1) were evaluated. A steady considerable decline in rrT1 with progressive reduction of liver function could be observed for both T1_in and T1_W (p < 0.001). When patients were subdivided into 3 different categories of ^13^C-MBT readouts, the groups could be significantly differentiated by their rrT1_in and rrT1_W values (p < 0.005). In a simple correlation model of ^13^C-MBT values with T1_inpost (r = 0.556; p < 0.001), T1_Wpost (r = 0.557; p < 0.001), rrT1_in (r = 0.711; p < 0.001) and rrT1_W (r = 0.751; p < 0.001), a log-linear correlation has been shown. Liver maximum capacity measured with ^13^C-MBT can be determined more precisely from gadoxetic acid-supported T1 mapping when intrahepatic fat is taken into account. Here, T1_W maps are shown to be significantly superior to T1_in maps without separation of fat.

## Introduction

For evaluation of liver function, medical professionals rely on a variety of diagnostic tools. A conservative method for evaluating liver function is the analysis of blood samples for hepatocytic transported organic anions (e.g., serum bilirubin), for serum enzymes detectable when the liver is injured (e.g., aminotransferases, alkaline phosphatase) or for liver’s biosynthetic capacity marker (e.g., serum proteins, albumin)^1^.

In recent years, the magnetic-resonance imaging (MRI), hepatocyte specific contrast agent gadoxetic acid (Gd-EOB-DTPA) has gained in importance, because T1 relaxometry supported by Gd-EOB-DTPA has proven to be a promising diagnostic method for assessing liver function or chronic hepatic diseases, in accordance with various clinical scoring systems (Child-Pugh score, MELD score), liver fibrosis scores (METAVIR score, ISHAK score), the ICG clearance test^2–7^ and only recently by our group with the ^13^C-methacetin breath test (^13^C-MBT)^8^. Gadoxetic acid is preferably imported into liver cells by respective ATP-dependent transporters (OATP1 B1/B3) during accumulation phase and excreted in the biliary system via multidrug resistance protein 2 (MRP2)^9,10^. Due to the paramagnetic properties of gadolinium, accumulation and increasing concentration of Gd-EOB-DTPA shortens T1 and to a lesser extent the T2 relaxation time, particularly that of water molecules and protons, allowing for the quantitative evaluation of liver function^6,11^. To allow for a “one-stop-shot” and in addition to conventional MRI-based information, such as the structure of liver parenchyma, liver vasculatures, liver lesions and their relation to vasculatures, gadoxetic acid-supported T1 mapping thus offers the potential to display imaging-based liver function. While established liver function tests such as the ICG-test or ^13^C-MBT, which enables evaluation of maximal liver function capacity (LiMax®) in real time^12,13^, only reflect global liver function, MRI-based assessment of liver function has the potential to illustrate both global and segmental level liver function.

In general, MRI signals in the human body are caused by hydrogen protons of water, triglycerides and fatty acids^14^. However, due to their characteristic chemical structure and properties, fatty acids exhibit a relatively short T1 relaxation time^15^; similarly, Gd-EOB-DTPA shortens the T1 relaxation time in interacting molecules. Therefore, in Gd-EOB-DTPA-supported T1 mapping, it still remains unclear to what extent the reduction of T1 relaxation time is induced by the presence of intrahepatic fat. It might therefore be assumed that the reduction rate and thus liver function are overestimated in patients suffering from non-alcoholic steatohepatitis (NASH) or fatty liver disease, as the T1 relaxation time is expected to decrease with increasing fat fraction and the relative voxel-specific uptake of contrast agent is diminished when intrahepatic fat is present.

In clinical practice, it is common to apply fat-suppression methods for diagnostic imaging to both improve visualization of pathology and enhance contrast. The Dixon method is an established tool for obtaining fat-suppressed water-only images or water-suppressed fat-only images^16–18^. In contrast to fat suppression by spectrally selective radiofrequency pulses, two contrasts at different echo times – typically in-phase and opposed-phase – are acquired, from which water and fat images can be calculated by a dedicated algorithm. This approach also has the advantage that the steady-state signal in a gradient echo sequence is not interrupted and all contrast including the derived water and fat images can be used for variable flip angle T1 mapping.

The purpose of this study was to compare in-phase, non-fat-suppressed T1 maps (T1_in) and water-only fat-suppressed T1 maps (T1_W) to evaluate the effect of intrahepatic fat on the MRI-based estimation of liver function.

## Results

### Patient characteristics

**Figure 1:**
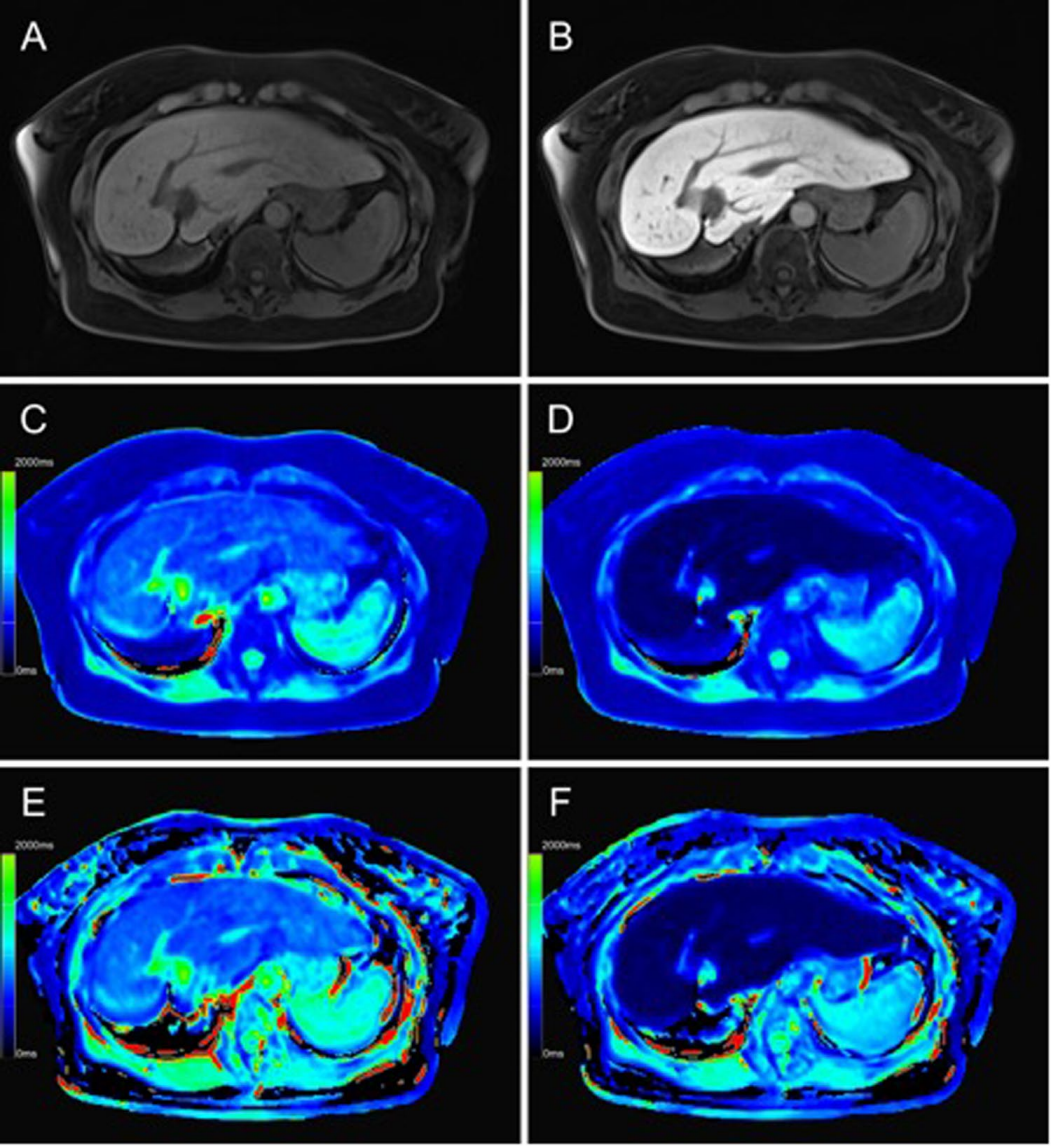
Color-coded T1 maps and T1-weighted sequence based on a 3D variable flip angle sequence of the liver parenchyma of a 47-year-old female patient with regular liver capacity (^13^C-MBT = 327 [µg/kg/h]). T1-weighted VIBE sequence with frequency-selective fat saturation (**A**,**B**) and T1 maps (**C**–**F**) were generated in native (**A**,**C**,**E**) and hepatobiliary phase (**B**,**D**,**F**). Respective T1-shortening effect can be observed as an increase in signal intensity of the liver in a T1-weighted VIBE sequence (**B**) and as a dark-blue region in color-coded T1 maps, representing a decreased relaxation time (**D**,**F**). Fat-water separation was applied according to the Dixon technique for the T1 maps (**E**,**F**). When applying fat suppression on T1 maps (**E**,**F**), the overall registered relaxation time increased compared with that of T1 maps without fat separation (**C**,**D**). The background of the T1 maps was manually set to black to facilitate color visualization.

**Figure 2:**
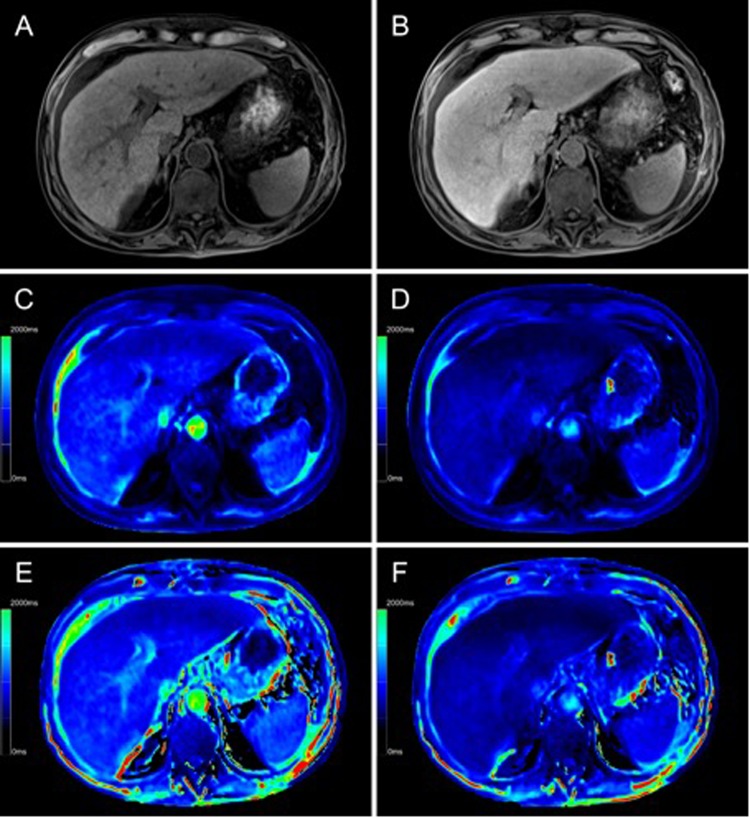
Color-coded T1 maps and T1-weighted sequence based on a 3D variable flip angle sequence of the liver parenchyma of a 63-year-old male patient with severe reduced liver capacity (^13^C-MBT = 135 [µg/kg/h]). T1-weighted VIBE sequence with frequency selective fat saturation (**A**,**B**) and T1 maps (**C–F**) were generated in native (**A**,**C**,**E**) and hepatobiliary phase (**B**,**D**,**F**). RespectiveT1-shortening effect can be observed as an increase in signal intensity of the liver in a T1-weighted VIBE sequence (**B**) and as a dark-blue region in color-coded T1 maps, representing a decreased relaxation time (**D**,**F**). Fat-water separation was applied according to the Dixon technique for the T1 maps (**E**,**F**). When applying fat suppression on T1 maps (**E**,**F**), the overall registered relaxation time increased compared with that of T1 maps without fat separation (**C**,**D**). The background of the T1 maps was manually set to black to facilitate color visualization.

A total of 79 patients (62 men and 17 women; mean age, 61.85 years; range, 39–82 years) were included in this study. All patients underwent a ^13^C-methacetin breath test (^13^C-MBT) within a timeframe of 24 h to a T1-weighted VIBE sequence with variable flip angles and Dixon water-fat separation. The ^13^C-MBT readout-dependent groups showed no significant difference in age, height and weight from each other, except for group 1, which was significantly shorter in height and lighter than groups 2 and 3 (p < 0.05; Table 1). Patients with a good ^13^C-MBT readout value also showed stronger contrast agent uptake during the hepatobiliary phase (Fig. 1) than did patients with a worse ^13^C-MBT readout value (Fig. 2).

**Table 1:** Patient characteristics.The different ^13^C-MBT readout groups did not differ significantly from each other with respect to age, height or weight. However, group 1 (>315.0 [µg/kg/h]) was significantly shorter (p < 0.05) and lighter (p < 0.05) than group 2 (140.0–315.0 [µg/kg/h]) and group 3 (<140.0 [µg/kg/h]).

parameter	n = 79	^13^C-MBT readout		
		>315.0 [µg/kg/h] n = 20	140.0–315.0 [µg/kg/h] n = 36	<140.0 [µg/kg/h] n = 23
age [years]	61.85 ± 9.54	61.00 ± 11.64	63.03 ± 9.83	60.74 ± 6.92
male	62 (78.48%)	8 (40.0%)	33 (91.67%)	21 (91.30%)
female	17 (21.52%)	12 (60.0%)	3 (8.33%)	2 (8.70%)
height [cm]	173.19 ± 7.65	168.70 ± 9.10	174.86 ± 7.36	174.48 ± 5.03
weight [kg]	86.08 ± 16.06	78.70 ± 21.64	89.08 ± 13.14	87.78 ± 12.98
rrT1_in [%]	50.05 ± 13.55	60.99 ± 7.66	52.21 ± 10.87	37.16 ± 11.06
rrT1_W [%]	51.75 ± 13.66	63.14 ± 6.72	54.04 ± 10.86	38.27 ± 10.87
^13^C-MBT [µg/kg/h]	227.38 ± 122.24	396.85 ± 60.25	217.53 ± 50.26	95.44 ± 36.75

All groups were significantly different with respect to rrT1_in and rrT1_W (p < 0.001).

Values indicate means ± standard deviation.

rrT1_in: reduction rate of T1 relaxation time using in-phase T1 mapping.

rrT1_W: reduction rate of T1 relaxation time using water-only T1 mapping.

^13^C-MBT: liver function capacity estimated by breath analysis of ^13^C-labeled methacetin metabolism.

### Dixon MR measurements compared to ^13^C-MBT readout

**Figure 3:**
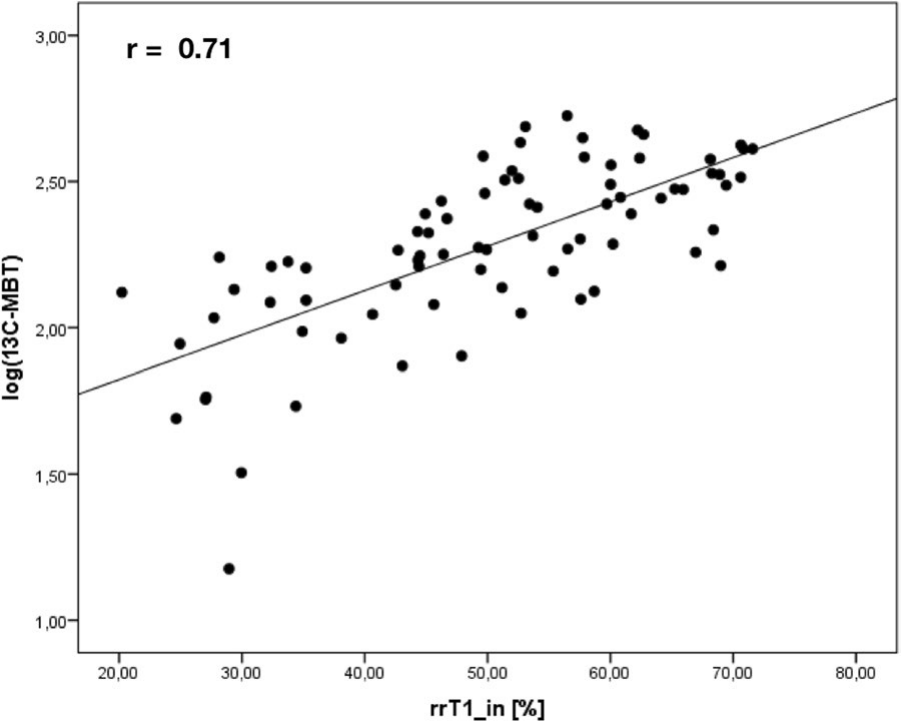
Correlation analysis of rrT1_in on logarithmic values of ^13^C-MBT readout (^13^C-MBT). Scatterplot of the reduction rate of T1 relaxation time in non-fat-saturated T1 maps (rrT1_in) in logarithmic values of ^13^C-MBT readouts.

**Figure 4:**
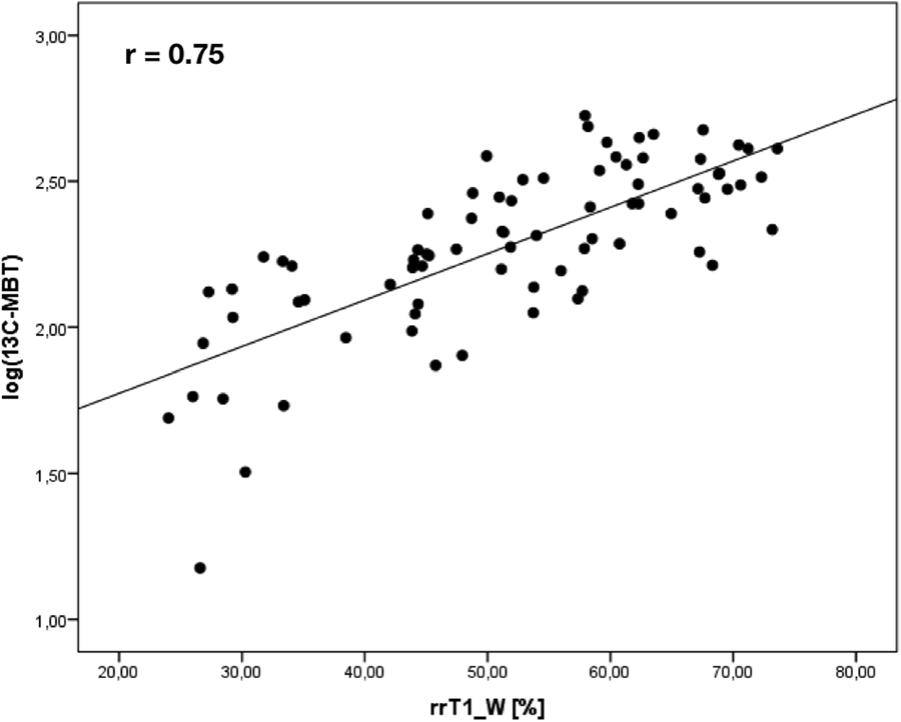
Correlation analysis of rrT1_W on logarithmic values of ^13^C-MBT readout (^13^C-MBT). Scatterplot of the reduction rate of T1 relaxation time in fat-saturated water-only T1 maps (rrT1_W), in logarithmic values of ^13^C-MBT readouts.

Considering all examinations, a simple linear regression model showed that both rrT1_in and rrT1_W (r = 0.711; r = 0.751; p < 0.001) are strong linear predictors of log-transformed ^13^C-MBT readouts (Table 2). When analyzing rrT1_in and rrT1_W data on a scatterplot of logarithmic values of ^13^C-MBT readout, both patterns showed strong similarities (Figs 3 and 4). A comparison of the correlation coefficients of rrT1_in and rrT1_W on ^13^C-MBT readouts reveals a significant difference (p = 0.0163).

**Table 2:** Simple linear regression models of rrT1_in and rrT1_W on ^13^C-MBT values.

		B (95%-CI)	P - value	r
Log(^13^C-MBT)	rrT1_in	0.015 (0.012; 0.019)	<0.001	0.711
	T1_in_post_	−0.001 (−0.002; −0.001)	<0.001	0.556
	rrT1_W	0.016 (0.013; 0.019)	<0.001	0.751
	T1_W_post_	−0.001 (−0.002; −0.001)	<0.001	0.557

rrT1_in: reduction rate of T1 relaxation time using in-phase T1 mapping.

rrT1_W: reduction rate of T1 relaxation time using water-only T1 mapping.

^13^C-MBT: liver function capacity estimated by breath analysis of ^13^C-labeled methacetin metabolism.

r: multiple correlation coefficient.

B: linear regression coefficient.

CI: confidence interval for B.

### Dixon MR measurements compared with group-specific ^13^C-MBT readout

In group 1 (normal liver function), the mean ^13^C-MBT readout was 396.85 ± 60.25 µg/kg/h, rrT1_in was 60.99 ± 7.66 and rrT1_W was 63.14 ± 6.72. The group with intermediate liver function (group 2) had a mean ^13^C-MBT readout of 217.53 ± 50.26 µg/kg/h, rrT1_in of 52.21 ± 10.87 and rrT1_W of 54.04 ± 10.86. In group 3, the observed mean ^13^C-MBT readout was 95.44 ± 36.75 µg/kg/h, whereas the mean rrT1_in was 37.16 ± 11.06 and the mean rrT1_W was 38.27 ± 10.87 (Table 1).

**Figure 5:**
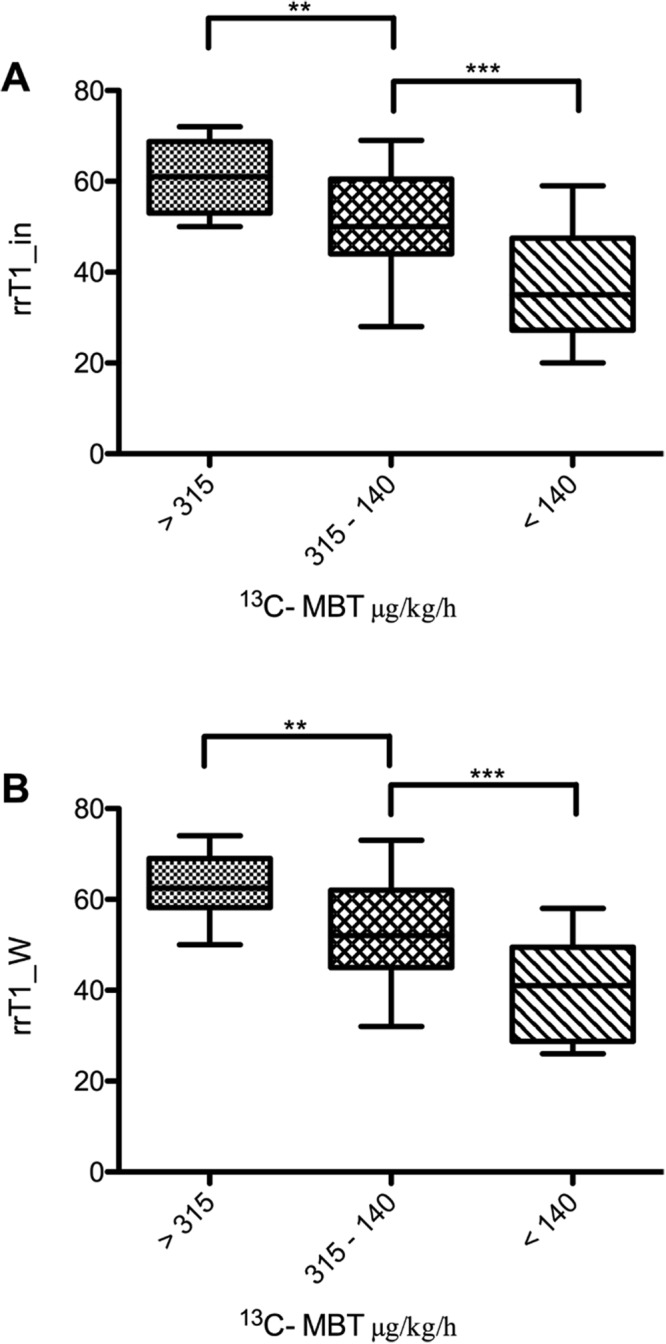
Reduction rates of T1 relaxation time by ^13^C-MBT readouts in T1 maps without fat separation (rrT1_in; **A**) and water-only T1 maps with fat separation (T1_W; **B**). The difference in reduction rates of the T1 relaxation time is statistically significant between the first and third groups in T1 maps without fat separation (A: p < 0.001) as well as in T1 maps with fat separation (B: p < 0.001). The difference is also statistically significant from that of the second group, either in T1 maps without fat separation (A: p = 0.003) or water-only T1 maps (B: p = 0.002). Group two differs significantly from group three in T1 maps without fat separation (A: p < 0.001) and T1 maps with fat separation (B: p < 0.001). group 1 ^13^C-MBT >315 [µg/kg/h]: normal liver capacity. group 2 ^13^C-MBT 315-140 [µg/kg/h]: intermediate liver capacity. group 3 ^13^C-MBT <140 [µg/kg/h]: severe impaired liver capacity. Data are shown as the mean T1 reduction rates ± standard deviation. **p < 0.01, ***p < 0.001.

All pairwise comparisons between groups 1 to 3 were statistically significant (p < 0.01) for both rrT1_in and rrT1_W (Fig. 5).

## Discussion

Nonalcoholic fatty liver disease (NAFLD) has been established as a serious disease for several decades. NAFLD is known to be accompanied by asymptomatic elevation of liver enzymes and nonalcoholic hypertransaminasemia^19^. Affected patients may experience a high risk for establishing insulin resistance, obesity, liver fibrosis, liver cirrhosis and hepatocellular carcinoma (HCC)^20^. The majority of patients with NAFLD have simple steatosis, whereas up to 30% suffer from non-alcoholic steatohepatitis (NASH) accompanied by an increasing risk of progression to end-stage liver disease^21^. The histological criterion for diagnosing NAFLD requires that 5% of hepatocytes contain intracellular fat^22^. As NAFLD-caused liver fibrotic cells contain a higher lipid content than do non-NAFLD-caused liver fibrotic cells, MR imaging might vary between the two.

Recently, gadoxetic acid-supported MR imaging was used to display liver function, as the intracellular uptake of Gd-EOB-DTPA and the consecutive shortening of the T1 relaxation time depend on hepatocyte function. Therefore, assessing imaging-based liver function when fat deposition in the liver varies is of clinical importance, as intrahepatic fat may either affect the measurement of hepatocyte-specific contrast agent uptake or affect the shortening of the T1 relaxation time itself due to its paramagnetic properties^7^. T1 relaxation indicates the rate of longitudinal relaxation, which depends on tissue type and can be altered by injection of paramagnetic materials. In common T1 mapping approaches, only one T1 value is considered per reconstructed voxel. This assumption may be violated in the presence of two different tissue types constituting two different compartments that contribute to the MR signal in a single voxel. In such a case, the determined value of T1 can be considered an effective T1 with a value in between that of the two tissue types. By using Dixon water-fat separation, the signals from water and fat compartments are separated before T1 calculation.

As the quantification properties of most tissues are known (e.g., macromolecules, small organic molecules, bound water, free water, and mobile fatty acids), tissues’ relaxation properties can be assumed, and a crude prediction of T1 and T2 relaxation values is possible^23^. The Dixon technique is a frequency-selective approach that achieves fat separation and quantification while processing the phase evolution differences of water and fat generated by different echo times (TEs). Therefore, in-phase and opposed-phase images of water and fat will be recorded, and water-only or fat-only images will be generated by an algorithm, respectively^16^.

As the Dixon method is a spectrally selective method and relaxation only affects the magnetization, independently of the resonance frequency, T1 mapping and Dixon method can be combined. Consequently, the T1 values of the separated species can be determined independently.

In almost all tissues, the detected T1 relaxation time per voxel is composed of the allocated T1 relaxation times of local fat and water protons; thus, the presence of intra-hepatic fat affects voxel evaluation. Voxels varying in partial volume of fat and hepatic tissue may lead to errors in T1 maps, which assume a single T1 species. Moreover, the hepatocyte-specific contrast agent Gd-EOB-DTPA is a nine-coordinate complex in which the EOB-DTPA-ligand occupies eight binding sites at the metal center of Gd^3+^ and the ninth coordination site is meant to be occupied by a solvent water molecule^11^. Due to dipole-dipole interactions with the paramagnetic contrast agent, the gadoxetic-supported T1 time of the water molecules will be shortened^11^, whereas respective T1 relaxation time of fatty acids may not or may only negligibly be affected.

Our analysis showed that Dixon-based water-only T1 maps correlate more strongly with the corresponding determined ^13^C-MBT (r = 0.751, p < 0.001) values than in-phase T1 maps without fat saturation (r = 0.711, p < 0.001; Table 2). Therefore, the elimination of fat (water-only T1 maps) improves the informative value for evaluation of liver function.

Similarly to the findings of Katsube *et al*.^6^, we could show that T1 mapping is a reliable tool for liver function estimation^3,8,24,25^. As the T1 relaxation time per voxel is affected by its fat and water composition and corresponding hepatic contrast agent uptake and T1 shortening, we assumed that diagnostic precision will change on water-only T1 maps relative to in-phase T1 maps.

The literature suggests that little is known about the effect of fat-suppressed T1 maps relative to that of non- fat suppressed maps. Papakonstantinou *et al*.^26^ compared fat-suppressed and conventional R2 relaxometry on hepatic, pancreatic, spleen and vertebral bone marrow tissue. The authors observed that fat suppression applied in a standard Carr-Purcell-Meiboom-Gill sequence improves the quantification of iron in lipid-rich tissues. Here, we used a different MRI sequence with water-only (fat-suppressed) and in-phase (not fat-suppressed) T1 relaxometry. However, similarly to Papakonstantinou *et al*., we were able to show that MRI-based estimation of liver function improves using a water-only sequence on T1 relaxometry maps. Several studies have examined either water-only or in-phase sequences on T1 maps to evaluate tissues functional status^27–30^. However, no group has compared the evaluative information quality of water-only T1 maps with that of in-phase T1 maps.

Nevertheless, our study has several restrictions: It was carried out with a small number of patients in only one study site and only one radiologist was involved in ROI placement. Due to the study’s retrospective design, we lacked histopathologic controls. Further prospective studies are required to identify patients’ fat fraction, which would enable the recruitment of a subset of patients suffering from non-alcoholic fatty liver disease.

In conclusion, our study showed that water-only T1 maps represent liver function, assessed by ^13^C-MBT, more accurately than do T1 maps without fat separation.

## Material and Methods

### Patients

The local institutional review board approval of the University Hospital Regensburg was obtained for this retrospective study. The study was performed in accordance with the relevant guidelines and regulations and written informed consent was obtained from all participating patients. The current retrospective analysis is based in parts on a previously published study of our group^8^.

Seventy-nine patients (62 men, 17 women; mean age 61.85 ± 9.54years) underwent both ^13^C-MBT (LiMAx® test) and gadoxetic acid-supported MRI at 3 T of the liver parenchyma with 3D variable flip angle T1 mapping combined with Dixon water-fat separation. Only patients without ^13^C-methacetin intolerance, a known reaction to gadoxetic acid or renal failure could be evaluated.

The underlying diseases for the MR examination and the ^13^C-MBT are summarized in Table 3.

**Table 3:** Underlying diseases and reasons for MRI examination.

		patients (n = 79)
HCC	with cirrhosis	46
	w/o cirrhosis	1
	liver cirrhosis	5
cholangiocarcinoma		5
benign liver lesion	focal nodular hyperplasia (FNH)	1
	hemangioma	3
secondary liver malignancies	carcinoma of the ileum or duodenum	2
	colon carcinoma	1
	mamma carcinoma	1
	rectal cancer	9
	sigma carcinoma	3
	thymoma	1
	uveal melanoma	1

The medical cases examined via MRI and ^13^C-MBT, divided according to their diseases.

### ^13^C-Methacetin breath test (^13^C-MBT)

Within 24 hours before or after the MRI examination, ^13^C-MBT was performed if patients fasted for at least 3 hours. Before 2 mg/kg body weight of ^13^C-methacetin could be administered intravenously, the baseline of normally exhaled ^12^CO2 was recorded over a period of 10 minutes. Finally, the change in the ^13^CO2/^12^CO2 ratio was measured over a period of 60 minutes and the amount of produced ^13^CO2 was determined. Based on the resulting ^13^C-MBT value[µg/kg/h], the patients could be divided into 3 different groups similar to our previous published study (normal liver function, >315; moderate liver function, 315-140; strongly reduced liver function, <140)^8,13,31^.

### MR imaging

A 3 T MANGETOM Skyra (Siemens Healthcare, Erlangen, Germany) with a combination of spine (32-channel spine matrix coil) and body array (18-channel body matrix coil) coil elements was used to generate T1 maps using T1- weighted images (VIBE-sequences) with Dixon water-fat separation (TE1/TE2: 2.46/3.69 ms; TR: 5.79 ms, reconstructed voxel size, 1.25 × 1.25 × 3.00 mm³; measured voxel size, 3.57 × 2.5 × 4.82 mm³; acquisition time, 17 sec) and multiple flip angles (1°, 7°, and 14°). Respective maps were generated prior to and 20 minutes after gadoxetic acid- administration in the hepatobiliary phase. Furthermore, the T1 mapping acquisitions were preceded by an additional B1 mapping sequence. These data were used in the inline T1 calculation to address B1 inhomogeneities that affect the determined T1 values. The patients received a body-weight-adapted gadoxetic acid dose (0.025 mmol/kg body weight) administered via bolus injection (flow rate, 1 mL/s) and 0.9% sodium chloride (20 mL) for flushing.

### Image analysis

An operator-defined region of interest (ROI) measurement was used to receive respective T1 relaxation times from maps in native and hepatobiliary phase. Here, 4 ROIs were manually placed with care at identical locations in respective maps excluding imaging artifacts, liver lesions or visible blood vessels. ROIs were circular and ranged from 0.8 to 4.4 cm² in size. The radiologists were blinded to the clinical, hematological and other radiological information to avoid measurement bias. The reduction rate of the T1 relaxation time (rrT1) was calculated according to the following formula^6,32^:

1 $${\rm{rrT}}1=|\frac{{\rm{T}}{1}_{{\rm{pre}}}-{\rm{T}}{1}_{{\rm{post}}}}{{\rm{T}}{1}_{{\rm{pre}}}}|\times 100\,( \% )$$

T1_pre_ and T1_post_ represent the T1 times before and after gadoxetic acid administration, respectively^8^.

### Statistical analysis

The different ^13^C-MBT readout groups were tested as non-parametric independent samples by the Mann-Whitney-U test. Gadoxetic acid-supported T1 mapping measurements (T1pre, T1post) and rrT1 (rrT1_in; rrT1_W) were tested in respective regression models to evaluate predictive power. Correlation coefficients were compared using the method proposed by Steiger. Statistical analyses were carried out using R (version 3.3.3., The R Foundation for Statistical Computing) and IBM SPSS Statistics (version 23, IL, USA) with a two-sided significance level of 0.05^8^.

## Data Availability

All data that support the findings of this study are provided in the manuscript. Raw data used in this work are available on reasonable request.
